# The roles of transmembrane 6 superfamily member 2 rs58542926 polymorphism in chronic liver disease: A meta‐analysis of 24,147 subjects

**DOI:** 10.1002/mgg3.824

**Published:** 2019-07-15

**Authors:** Xinpei Chen, Pengcheng Zhou, Luo De, Bo Li, Song Su

**Affiliations:** ^1^ Department of Hepatobiliary Surgery The Affiliated Hospital of Southwest Medical University Luzhou China

**Keywords:** chronic liver disease, meta‐analysis, rs58542926 polymorphisms, transmembrane 6 superfamily member 2 (*TM6SF2*)

## Abstract

**Background:**

Some genetic association studies tried to investigate potential associations of transmembrane 6 superfamily member 2 (*TM6SF2*) polymorphisms with chronic liver disease. However, the results of these studies were not consistent. Thus, we performed the present meta‐analysis to explore associations between *TM6SF2* polymorphisms and chronic liver disease in a larger pooled population.

**Methods:**

Systematic literature research of PubMed, Web of Science, Embase, and CNKI was performed to identify eligible studies for pooled analyses. I^2^ statistics were employed to assess between‐study heterogeneities. If I^2^ was greater than 50%, random‐effect models (REMs) would be used to pool the data. Otherwise, fixed‐effect models (FEMs) would be applied for synthetic analyses.

**Results:**

Totally 28 studies were included for analyses (13,137 cases and 11,010 controls). The pooled analyses showed that rs58542926 polymorphism was significantly associated with chronic liver disease in overall population (dominant model: *p* < 0.0001, OR = 0.70, 95% CI = 0.64–0.76, I^2^ = 47%; recessive model: *p* < 0.0001, OR = 2.94, 95% CI = 2.05–4.20, I^2^ = 0%; over‐dominant model: *p* < 0.0001, OR = 1.34, 95% CI = 1.23–1.47, I^2^ = 0%; allele model: *p* < 0.0001, OR = 0.68, 95% CI = 0.63–0.73, I^2^ = 47%), and these significant findings were further confirmed in both Asians and Caucasians. Stratified analyses by type of disease revealed similar positive results in hepatocellular carcinoma (HCC), cirrhosis, alcoholic liver disease (ALD) and NAFLD (Nonalcoholic fatty liver disease), but not in chronic hepatitis B infection (CHB) and chronic hepatitis C infection (CHC).

**Conclusions:**

These results suggested that *TM6SF2* rs58542926 could be used to identify individuals at higher susceptibility to chronic liver disease, especially for HCC, cirrhosis, ALD, and NAFLD.

## 1 INTRODUCTION

Chronic liver disease is a major global health threat and it currently accounts for approximately 3.5% of all deaths worldwide (Asrani, Devarbhavi, Eaton, & Kamath, 2019). Cirrhosis and hepatocellular carcinoma (HCC) are the two leading causes of liver‐related deaths. Annually, cirrhosis causes 1.16 million deaths, and HCC causes 788,000 deaths, making them the 11th and 16th most common causes of death respectively (Marcellin & Kutala, 2018; Peery et al., 2019). The etiologies of chronic liver disease are highly complex. Although excessive alcohol intake, obesity, and chronic viral infection were already verified to be pathogenic factors of different types of chronic liver disease (Lee et al., 2012; Saracco et al., 2016), the fact that the likelihoods of developing chronic liver disease in these exposed to above mentioned etiological factors were quite different suggested that genetic factors also played crucial parts in the pathogenesis of chronic liver disease.

The transmembrane 6 superfamily 2 (*TM6SF2*) gene is responsible for regulating lipid metabolism in the liver. Previous experimental studies demonstrated that *TM6SF2* siRNA inhibition was associated with a reduced secretion of triglyceride‐rich lipoproteins and an increased triglyceride aggregation in hepatocytes, whereas TM6SF2 overexpression was associated with reduced liver cell steatosis (Li et al., 2018; Mahdessian et al., 2014). Recently, two genome‐wide association studies conducted by Kozlitina et al. (2014) and Liu et al. (2014) found that the transmembrane 6 superfamily member 2 (*TM6SF2*) rs58542926 polymorphism (a functional variant that was associated with altered gene expression levels) was not only associated with higher liver fat levels, but also associated with elevated serum levels of alanine transaminase and advanced liver fibrosis, supporting that this polymorphism might play crucial roles in the development of different types of liver diseases. Since then, several genetic association studies were performed in diverse populations to estimate potential associations between rs58542926 polymorphism and chronic liver disease, with inconsistent results (Bale et al., 2017; Krawczyk et al., 2017; Manchiero et al., 2017; Milano et al., 2015). Therefore, we conducted a meta‐analysis of all relevant studies to more comprehensively analyze the effects of *TM6SF2* polymorphisms on individual susceptibility to chronic liver disease in a larger pooled population.

## 2 MATERIALS AND METHODS

We reported this meta‐analysis as suggested by the Reporting Items for Systematic Reviews and Meta‐analyses (PRISMA) guideline (Moher, Liberati, Tetzlaff, Altman, & PRISMA group, 2009).

### 2.1 Literature search and inclusion criteria

Eligible studies were retrieved from PubMed, Web of Science, Embase, and CNKI using the following searching strategy: (transmembrane 6 superfamily member 2 OR TM6SF2) AND (polymorphism OR variant OR variation OR mutation OR genome‐wide association study OR genetic association study) AND (chronic liver disease OR chronic hepatitis OR HBV OR HCV OR nonalcoholic fatty liver disease OR nonalcoholic fatty liver OR nonalcoholic steatohepatitis OR alcoholic liver disease OR liver cirrhosis OR hepatocellular carcinoma). The initial search was conducted in November 2018 and the latest update was performed in February 2019. Moreover, we also screened the references of all retrieved articles to identify other potential relevant studies.

Included studies must satisfy the following criteria: (a) genetic association studies about *TM6SF2* polymorphisms and chronic liver disease in human beings; (b) provide genotypic/allelic frequency of investigated polymorphisms in cases and controls; (c) full text in English or Chinese available. Studies were excluded if one of the following criteria was fulfilled: (a) not about *TM6SF2* polymorphisms and chronic liver disease; (b) studies that were not performed in human beings; (c) case reports or case series; (d) reviews, comments, and conference presentations.

### 2.2 Data extraction and quality assessment

We extracted following data from included studies: the name of the first author, publication year, country and ethnicity of study subjects, sample size, and genotypic/allelic distributions of *TM6SF2* polymorphisms in cases and controls. The probability value (*p* value) of Hardy–Weinberg equilibrium (HWE) was also calculated. When necessary, we wrote to the corresponding authors for extra information. We used the Newcastle‐Ottawa scale (NOS) to assess the quality of eligible studies (Stang, 2010). This scale has a score range of zero to nine, and studies with a score of more than seven were thought to be of high quality. Data extraction and quality assessment were performed by two independent reviewers. Any disagreement between two reviewers was solved by discussion until a consensus was reached.

### 2.3 Statistical analyses

We used Review Manager Version 5.3.3 (The Cochrane Collaboration, Software Update) to conduct statistical analyses. We calculated odds ratios (ORs) and 95% confidence intervals (CIs) to estimate strength of associations in all possible genetic models, and statistical significances of pooled analyses were determined by the Z test, with a *p* value of 0.05 or less was defined as statistically significant. I^2^ statistics were employed to assess between‐study heterogeneities. If I^2^ was greater than 50%, random‐effect models (REMs) would be used to pool the data on account of significant heterogeneities. Otherwise, fixed‐effect models (FEMs) would be used for synthetic analyses. Subgroup analyses by ethnicity of participants and type of disease were performed. Stabilities of synthetic results were evaluated with sensitivity analyses, and publication biases were evaluated with funnel plots.

## 3 RESULTS

### 3.1 Characteristics of included studies

**Figure 1:**
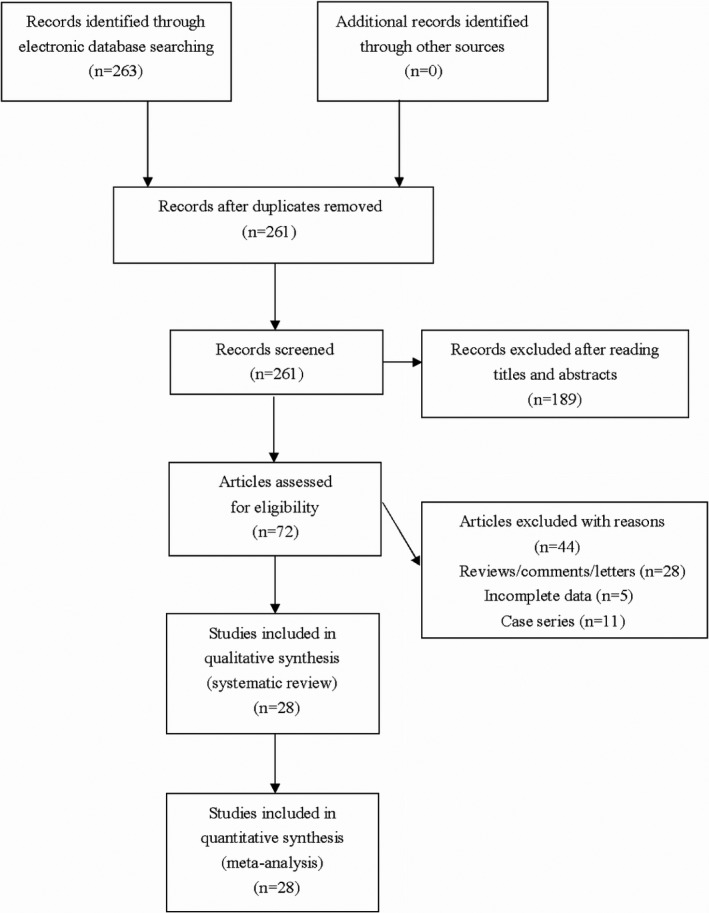
Flowchart of study selection for the present study

The initial literature search found 263 potential relevant articles. Among these articles, totally 28 studies met the inclusion criteria and thus were included for pooled analyses (see Figure 1). The NOS score of eligible articles ranged from 7 to 8, which indicated that all included studies were of high quality. Baseline characteristics of included studies were shown in Table 1.

**Table 1:** The characteristics of included studies for *TM6SF2* rs58542926 polymorphism and chronic liver disease

First author (year)	Country	Ethnicity	Type of disease	Sample size	Genotype distribution		*P*‐value for HWE	NOS score
					Controls	Controls		
Bale (2017)	India	Mixed	NAFLD	250/232	171/66/13	190/40/2	0.947	8
Buch (2015)	Germany	Caucasian	ALD	712/1426	NA	NA	NA	7
Coppola (2015)	Italy	Caucasian	Cirrhosis	101/47	85/16/0	45/2/0	0.882	7
Donati (2017)	Italy	Caucasian	HCC	132/633	109/19/4	538/88/7	0.121	8
Eslam (2016)	Australia	Caucasian	CHB	507/228	450/55/2	197/30/1	0.901	8
Eslam (2016)	Australia	Caucasian	CHC	2023/228	1778/235/10	197/30/1	0.901	8
Eslam (2016)	Australia	Caucasian	NAFLD	502/228	391/100/11	197/30/1	0.901	8
Falleti (2016)	Italy	Caucasian	Cirrhosis	511/228	443/66/2	203/25/0	0.381	8
Falleti (2016)	Italy	Caucasian	CHB + CHC	285/228	255/30/0	203/25/0	0.381	8
Falleti (2016)	Italy	Caucasian	ALD	226/228	188/36/2	203/25/0	0.381	8
Falleti (2016)	Italy	Caucasian	NAFLD	150/228	123/26/1	203/25/0	0.381	8
Goffredo (2016)	Italy	Caucasian	NAFLD	158/296	135/22/1	270/26/0	0.429	7
Grove (2018)	UK	Caucasian	NAFLD	186/439	NA	NA	NA	7
Jiang (2018)	China	Asian	CHB	288/106	254/33/1	96/10/0	0.610	8
Koo (2018)	Korea	Asian	NAFLD	365/96	306/57/2	87/9/0	0.630	7
Krawczyk (2016)	Germany	Caucasian	NAFLD	143/180	110/30/3	152/28/0	0.258	8
Krawczyk (2016)	Germany	Caucasian	Cirrhosis	90/205	68/20/2	164/38/3	0.641	8
Kruk (2018)	Poland	Caucasian	Cirrhosis	55/123	50/5/0	107/16/0	0.440	7
Li (2019)	China	Asian	NAFLD	201/239	188/13/0	234/5/0	0.870	8
Liu (2014)	UK	Caucasian	NAFLD	349/379	271/70/8	328/49/2	0.908	8
Manchiero (2017)	Brazil	Mixed	Cirrhosis	58/232	46/12/0	215/17/0	0.562	7
Milano (2015)	Italy	Caucasian	CHC	815/231	746/69/0	201/29/1	0.966	8
Musso (2017)	Italy	Caucasian	NAFLD	60/60	37/21/2	40/20/0	0.121	7
Raksayot (2019)	Thailand	Asian	CHB	270/105	218/51/1	89/15/1	0.682	8
Raksayot (2019)	Thailand	Asian	CHC	131/105	101/29/1	89/15/1	0.682	8
Raksayot (2019)	Thailand	Asian	HCC	132/105	78/46/8	89/15/1	0.682	8
Sagnelli (2016)	Italy	Caucasian	Cirrhosis	31/136	26/5/0	124/12/0	0.590	7
Sookoian (2015)	Argentina	Mixed	NAFLD	226/135	184/37/5	120/15/0	0.494	8
Stickel (2018)	Switzerland	Caucasian	HCC	751/1165	558/164/29	957/193/15	0.143	7
Teng (2018)	China	Asian	CHB	160/179	142/18/0	161/18/0	0.479	8
Teng (2018)	China	Asian	Cirrhosis	239/179	209/29/1	161/18/0	0.479	8
Wang (2018)	China	Asian	NAFLD	367/366	302/65/0	333/33/0	0.366	8
Wang (2016)	China	Asian	CHB	683/364	608/73/2	331/33/0	0.365	8
Wang (2016)	China	Asian	Cirrhosis	677/364	602/74/1	331/33/0	0.365	8
Wang (2016)	China	Asian	HCC	418/364	363/55/0	331/33/0	0.365	8
Xu (2018)	China	Asian	CHB	260/156	229/30/1	140/16/0	0.500	8
Yue (2018)	China	Asian	NAFLD	118/122	111/7/0	114/8/0	0.708	7
Zhang (2018)	China	Asian	ALD	507/645	435/65/7	559/83/3	0.966	8

Abbreviations: TM6SF2, Transmembrane 6 superfamily 2; HCC, Hepatocellular carcinoma; NAFLD, Nonalcoholic fatty liver disease; ALD, Alcoholic liver disease; CHB, Chronic hepatitis B infection; CHC, Chronic hepatitis C infection; HWE, Hardy–Weinberg equilibrium; NOS, Newcastle‐Ottawa scale; NA, Not available.

### 3.2 Overall and subgroup analyses

The results of overall and subgroup analyses were summarized in Table 1. To be brief, 13,137 cases and 11,010 controls were eligible for analyses, the pooled analyses showed that rs58542926 polymorphism was significantly associated with chronic liver disease in overall population (dominant model: *p* < 0.0001, OR = 0.70, 95% CI = 0.64–0.76, I^2^ = 47%; recessive model: *p* < 0.0001, OR = 2.94, 95% CI = 2.05–4.20, I^2^ = 0%; over‐dominant model: *p* < 0.0001, OR = 1.34, 95% CI = 1.23–1.47, I^2^ = 0%; allele model: *p* < 0.0001, OR = 0.68, 95% CI = 0.63–0.73, I^2^ = 47%), and these significant findings were also confirmed in both Asians (dominant, recessive, over‐dominant, and allele models) and Caucasians (dominant, recessive, over‐dominant, and allele models). Further stratified analyses by type of disease revealed similar positive results in hepatocellular carcinoma (HCC), cirrhosis, alcoholic liver disease (ALD), and NAFLD (Nonalcoholic fatty liver disease), but not in chronic hepatitis B infection (CHB) and chronic hepatitis C infection (CHC) (see Table 2).

**Table 2:** Results of pooled analyses for *TM6SF2* rs58542926 polymorphism and chronic liver disease

Population	Sample size	Dominant comparison			Recessive comparison			Overdominant comparison			Allele comparison		
		*p* value	OR (95%CI)	I^2^ statistic	*p* value	OR (95%CI)	I^2^ statistic	*p* value	OR (95%CI)	I^2^ statistic	*p* value	OR (95%CI)	I^2^ statistic
Overall	13137/11010	**<0.0001**	**0.70 (0.64–0.76)**	47%	**<0.0001**	**2.94 (2.05–4.20)**	0%	**<0.0001**	**1.34 (1.23–1.47)**	0%	**<0.0001**	**0.68 (0.63–0.73)**	47%
Asian	4816/3495	**<0.0001**	**0.69 (0.60–0.79)**	34%	**0.03**	**2.29 (1.07–4.87)**	0%	**<0.0001**	**1.41 (1.22–1.63)**	28%	**<0.0001**	**0.69 (0.60–0.79)**	28%
Caucasian	7787/6916	**0.004**	**0.77 (0.64–0.92)**	52%	**<0.0001**	**2.85 (1.85–4.37)**	0%	**0.0002**	**1.25 (1.11–1.40)**	39%	**<0.0001**	**0.74 (0.63–0.86)**	54%
HCC	1433/2267	**0.003**	**0.58 (0.40–0.83)**	65%	**<0.0001**	**3.29 (1.92–5.66)**	0%	**0.01**	**1.55 (1.10–2.18)**	59%	**0.0007**	**0.58 (0.42–0.79)**	62%
Cirrhosis	1762/1514	**0.006**	**0.72 (0.57–0.91)**	25%	0.37	1.79 (0.50–6.37)	0%	**0.01**	**1.36 (1.08–1.71)**	27%	**0.004**	**0.73 (0.58–0.90)**	18%
NAFLD	3075/3000	**<0.0001**	**0.54 (0.46–0.64)**	0%	**<0.0001**	**5.20 (2.43–11.13)**	0%	**<0.0001**	**1.69 (1.44–1.98)**	0%	**<0.0001**	**0.55 (0.48–0.63)**	0%
ALD	1445/2299	0.18	0.82 (0.62–1.10)	40%	0.06	3.33 (0.97–11.44)	0%	0.41	1.13 (0.84–1.51)	42%	**<0.0001**	**0.71 (0.60–0.84)**	26%
CHB	2168/1138	0.34	0.89 (0.71–1.12)	0%	0.84	1.14 (0.33–3.92)	0%	0.38	1.11 (0.88–1.40)	0%	0.31	0.89 (0.72–1.11)	0%
CHC	2969/564	0.44	0.60 (0.16–2.19)	65%	0.71	1.10 (0.68–1.77)	0%	0.75	0.93 (0.58–1.49)	63%	0.65	0.90 (0.57–1.41)	64%

Note

The values in bold represent there is statistically significant differences between cases and controls.

Abbreviations: HCC, Hepatocellular carcinoma; ALD, Alcoholic liver disease; NAFLD, Nonalcoholic fatty liver disease; CHB, Chronic hepatitis B infection; CHC, Chronic hepatitis C infection; OR, Odds ratio; CI, Confidence interval; NA, Not available.

### 3.3 Sensitivity analyses

We performed sensitivity analyses to test the effects of individual study on pooled results. No any altered results were observed in overall and subgroup comparisons, which indicated that our findings were statistically robust.

### 3.4 Publication biases

We used funnel plots to assess publication biases. We did not find obvious asymmetry of funnel plots in any comparisons, which suggested that our findings were unlikely to be impacted by severe publication biases (see Figure S1).

## 4 DISCUSSION

As far as we know, this is to date the first meta‐analysis on associations of *TM6SF2* polymorphisms with chronic liver disease, and our pooled analyses suggested that rs58542926 polymorphism was significantly associated with chronic liver disease in both Asians and Caucasians. Further stratified analyses revealed similar positive results in HCC, cirrhosis, ALD and NAFLD, but not in CHB and CHC.

There are several notable points about this meta‐analysis. Firstly, although several different types of chronic liver disease were combined for analyses, between‐study heterogeneities in overall analyses were only mild, which suggested that pool the results of these studies was feasible. Secondly, subgroup analyses by type of disease suggested that the positive results were mainly driven by HCC, cirrhosis, ALD, and NAFLD. However, it is worth noting that the trends of associations for CHB and CHC were identical to that for overall analyses. Considering that the sample sizes of pooled analyses with regard to the ALD, CHB, and CHC were still relatively small. It is possible that our study was still not statistically adequate to detect the actual associations between rs58542926 polymorphism and these liver diseases. Therefore, further studies with larger sample sizes still need to test the associations between rs58542926 polymorphism and chronic liver disease, especially for ALD, CHB and CHC. Thirdly, the pathogenesis of chronic liver disease is extremely complex, and therefore the probability that a specific genetic polymorphism could significantly contribute to its development is low, and we strongly recommend further studies to perform haplotype analyses and explore potential gene‐gene interactions. Fourthly, to more precisely measure the effects of certain endogenous/exogenous factors on disease occurrence and development, molecular pathologic epidemiology (MPE) analyses should be adopted. However, since included studies only focused on the effects of rs58542926 polymorphism on individual susceptibility to chronic liver disease, such analyses were not applicable in the current meta‐analysis. But to better elucidate the underlying pathogenesis mechanisms of chronic liver disease, future studies should try to investigate the interaction of rs58542926 polymorphism (as endogenous factors) with potential pathogenic environmental factors (as exogenous factors) as an MPE approach (Nishi et al., 2016). Fifthly, the present meta‐analysis aimed to explore associations between all *TM6SF2* polymorphisms and chronic liver disease. However, only rs58542926 polymorphism could be analyzed in the current study because no any other *TM6SF2* polymorphisms were investigated by at least two different genetic association studies.

Like all meta‐analysis, this study certainly has some limitations. First, due to lack of raw data, adjusted analyses were inapplicable, and we have to admit that failure to perform further adjusted analyses for potential confounding factors might impact the reliability of our findings (Zhang, Guo, Qin, & Li, 2016; Zhang, Zhu, Huo, Qin, & Yuan, 2016). Second, associations between rs58542926 polymorphisms and chronic liver disease might also be modified by gene‐environmental interactions. However, we could not perform relevant analyses accordingly since most of studies did not investigate these associations (Abdel‐Hamed, Ghattas, Mesbah, Saleh, & Abo‐Elmatty, 2017; Zhang, Guo, et al., 2016; Zhang, Zhu, et al., 2016). Third, gray literatures that were not formally published in academic journals were not considered to be eligible for analyses in this meta‐analysis since it is hard to determine their quality. However, since gray literatures were not analyzed, although funnel plots suggested that severe publication biases were unlikely, it is still possible that our findings may be impacted by potential publication biases (Kapil et al., 2016). On account of above mentioned limitations, our findings should be cautiously interpreted.

In conclusion, our meta‐analysis suggested that *TM6SF2* rs58542926 polymorphism might affect individual susceptibility to chronic liver disease in both Asians and Caucasians. Further stratified analyses revealed similar positive results in HCC, cirrhosis and NAFLD, but not in ALD, CHB, and CHC. These results suggested that this polymorphism could be used to identify individuals at higher susceptibility to chronic liver disease, especially for HCC, cirrhosis, and NAFLD. Future investigations are warranted to explore potential roles of other *TM6SF2* polymorphisms in the development of chronic liver disease.

## CONFLICT OF INTEREST

The authors declare that they have no conflict of interest.

## AUTHORS' CONTRIBUTIONS

Xinpei Chen, Pengcheng Zhou, De Luo, and Song Su conceived of the study, participated in its design. Xinpei Chen, Pengcheng Zhou, and De Luo conducted the systematic literature review. Bo Li performed data analyses. Xinpei Chen, Pengcheng Zhou, De Luo, and Song Su drafted the manuscript. All gave final approval and agree to be accountable for all aspects of work ensuring integrity and accuracy.
