# Supplementary material for: The roles of transmembrane 6 superfamily member 2 rs58542926 polymorphism in chronic liver disease: A meta‐analysis of 24,147 subjects
Source: Mol Genet Genomic Med. 2019 Jul 15;7(8):e824. doi: 10.1002/mgg3.824 (PMC6687636; doi:10.1002/mgg3.824)
Supplement: Supplementary file 1 [file MGG3-7-e824-s001.docx]

**Supplementary Fig. 1. Funnel plots of overall analyses**


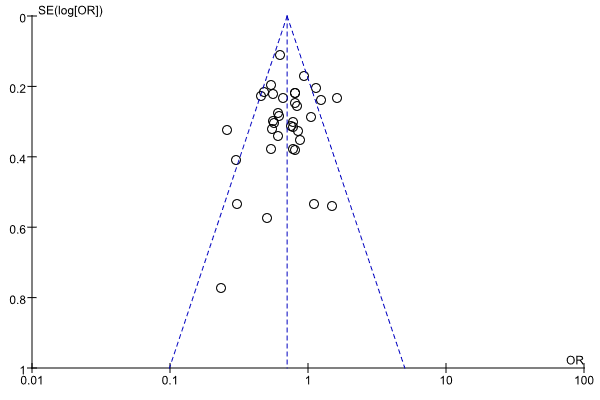


Funnel plot of TM6SF2 rs58542926 polymorphism and chronic liver disease under dominant comparison


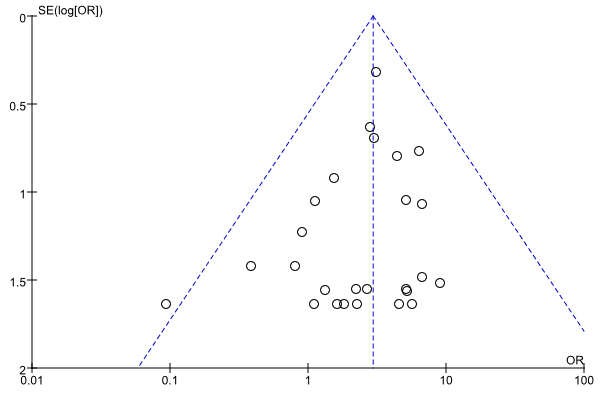


Funnel plot of TM6SF2 rs58542926 polymorphism and chronic liver disease under recessive comparison


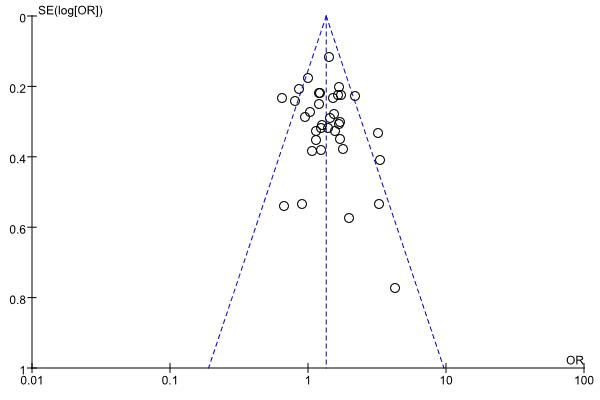


Funnel plot of TM6SF2 rs58542926 polymorphism and chronic liver disease under overdominant comparison


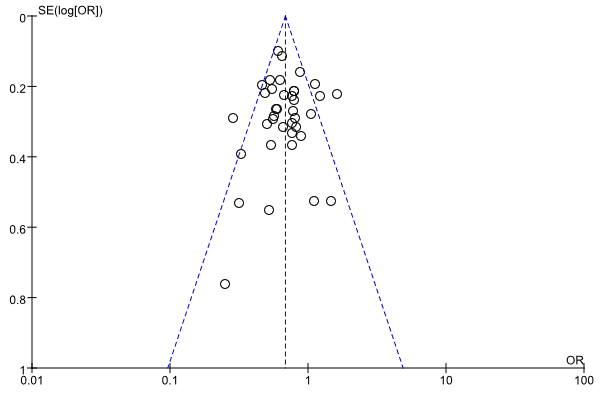


Funnel plot of TM6SF2 rs58542926 polymorphism and chronic liver disease under allele comparison
